# Candidate tumour suppressor CCDC19 regulates miR-184 direct targeting of C-Myc thereby suppressing cell growth in non-small cell lung cancers

**DOI:** 10.1111/jcmm.12317

**Published:** 2014-06-26

**Authors:** Zhen Liu, Chunping Mai, Huiling Yang, Yan Zhen, Xiaoli Yu, Shengni Hua, Qiangyun Wu, Qinping Jiang, Yajie Zhang, Xin Song, Weiyi Fang

**Affiliations:** a Department of Pathology, School of Basic Medicine, Guangzhou Medical UniversityGuangzhou, China; b Cancer Research Institute, Southern Medical UniversityGuangzhou, China; c School of Pharmacy, Guangdong Medical CollegeDongguan, China; d Third Affiliated Hospital of Guangzhou Medical CollegeGuangzhou, China; e Department of Cancer Biotherapy Center, Third Affiliated Hospital of Kunming Medical UniversityKunming, China

**Keywords:** CCDC19, tumour suppressor, lung cancer, PI3K/AKT, miR-184

## Abstract

We previously reported and revised the nasopharyngeal epithelium specific protein CCDC19 and identified it as a potential tumour suppressor in nasopharyngeal carcinoma. The purpose of this study was to investigate the involvement of CCDC19 in the pathogenesis of human non-small cell lung cancers (NSCLC). Down-regulated CCDC19 expression was observed in NSCLC tissues and cells compared to normal tissues. However, reduced protein expression did not correlate with the status of NSCLC progression. Instead, we observed that patients with lower CCDC19 expression had a shorter overall survival than did patients with higher CCDC19 expression. Lentiviral-mediated CCDC19 overexpression significantly suppressed cell proliferation and cell cycle transition from G1 to S and G2 phases in NSCLC cells. Knocking down CCDC19 expression significantly restored the ability of cell growth in CCDC19 overexpressing NSCLC cells. Mechanistically CCDC19 functions as a potential tumour suppressor by stimulating miR-184 suppression of C-Myc thus blocking cell growth mediated by the PI3K/AKT/C-Jun pathway. Our studies are the first to demonstrate that reduced expression of CCDC19 is an unfavourable factor in NSCLC.

## Introduction

Worldwide, lung cancer is the most common cause of cancer-related death in men and women and is responsible for 1.38 million deaths annually as of 2008 [1]. Non-small cell lung cancers (NSCLC) are the predominant types of lung cancer with low 5-year survival rate. Although various therapeutic methods have been developed, most patients die because of an insufficient recognition of the complicated pathogenesis of NSCLC. Synergetic effects of genetic alterations, smoking and environmental factors are thought to drive abnormal gene expression, which contribute to the initiation and development of NSCLC [2–5].

CCDC19 was originally isolated from normal human nasopharynx mucosa using differential display (Accession number: NM_012337.1; Official name: CCDC19) and is specifically expressed in human nasopharynx and trachea [6]. In a subsequent investigation, we revised the open reading frame (ORF) sequence of CCDC19 and updated its version number to NM_012337.2 in the NCBI GeneBank database. Further, we observed that CCDC19 acted as a potential tumour suppressor inhibiting cell growth, migration and invasion by modulating the cell cycle and MAPK pathway in nasopharyngeal carcinoma (NPC) [7,8]. However, the expression pattern and functional mechanisms of CCDC19 in lung cancer have not been reported.

miR-184 is evolutionarily conserved at the nucleotide level from flies to humans [9]. It is located within region on the 15q25.1 in humans and its corresponding transcript is comparatively small (84 bp) which is not encoded near other clustered miRNAs [10]. Recently, miR-184 has been implicated in the pathogenesis of several types of tumour and plays dual roles in different tumours. It is observed to be significantly increased in tongue and liver tumour tissues in comparison with their respective normal tissues and acts as a potential oncogene in these tumours [11,12]. On the contrary, miR-184 is found to be down-regulated in renal carcinoma [13] and acts as a candidate tumour suppressor in neuroblastoma [14]. However, the role of miR-184 is still to be determined in other tumours.

In this study, we present evidence that CCDC19 as a potential tumour suppressor, activates the expression of miR-184. The latter further directly suppresses the expression of C-Myc, a key oncogenic transcription factor inducing the pathogenesis of tumours [15,16], participating in the inhibition of CCDC19-mediated cell cycle transition and the growth of NSCLC. Our findings provide a deeper understanding of the anti-cancer mechanisms of CCDC19.

## Materials and methods

### Cell culture, tissue collection and ethics statement

Lung cancer cell lines including A549, SPCA-1, H446, 95D and H460 were maintained in RPMI 1640 supplemented with 10% newborn calf serum (NBCS; PAA Laboratories, Inc, Pasching, Austria). Freshly isolated primary lung tissues (three cases for squamous cell cancers and three cases for adenocarcinoma), seven freshly isolated lung tissues, 73 paraffin-embedded undifferentiated NSCLC (squamous cell cancer and adenocarcinoma) specimens and 22 paraffin-embedded non-cancerous lung tissues were obtained at the time of diagnosis before any therapy from The First Affiliated Hospital of Guangzhou Medical University, Guangzhou, and Tumor Hospital of Yunnan Province, Kunming, China. In the 73 lung cancer cases, there were 53 male and 20 female with ages ranging from 19 to 85 years (median, 59.37 years). The clinical follow-up time of patients ranged from 1 to 50 months. For the use of these clinical materials for research purposes, prior written informed consents from all the patients and approval from the Ethics Committees of Tumor Hospital of Yunnan Province were obtained. All specimens had confirmed pathological diagnosis and were staged according to the 2010 lung cancer staging system of the AJCC [17].

### Western blot

Western blotting was performed with a SDS-PAGE Electrophoresis System according to previous reports [7,13] with the following antibodies: rabbit polyclonal CCDC19 (Proteintech Group, Inc. Chicago, IL, USA), ACTB, GAPDH, CCND1, p21, E2F1, p15 (Santa Cruz Biotechnology, Santa Cruz, CA, USA), C-Myc, C-Jun, AKT, p-Akt (Ser-473), PI3K, p16, p27, E-Cadherin and pPI3K (Tyr458; 1:1000; Cell Signaling Technology, Danvers, MA, USA). Signals were detected using enhanced chemiluminescence reagents (Pierce, Rockford, IL, USA).

### Immunohistochemistry

Examination of CCDC19 expression in samples of lung cancer and normal tissues by IHC was performed as previously described [7,8]. Stained tissue sections were reviewed and scored independently by two pathologists blinded to the clinical parameters. The staining score standard has also been described [7,8]. For statistical analysis of CCDC19 expression in non-cancerous tissues against lung cancer tissues, the staining score of 0–4 and 5–6 were respectively considered to be low or high expression.

### Establishment of lentivirus-delivered CCDC19-overexpressed in NSCLC cells

Construction of lentivirus pGC-FU-CCDC19-GFP vector and lentiviral packaging have been reported in previous studies [7,8]. The lentiviral particles were used to infect NSCLC cell lines A549 and SPAC1 and the levels of CCDC19 protein were measured using Western blot assays.

### Transient transfection with siRNAs for CCDC19

Small-interfering RNAs (siRNAs) for CCDC19 and negative control sequences were designed and synthesized (Table S1) by Guangzhou RiboBio (RiboBio Inc, Guangzhou, China). Twenty-four hours prior to transfection, lung cancer cells A549 and SPAC1 with CCDC19 overexpression were plated onto a 96-well or 6-well plates (Nest, Biotech, Hongkong, China) at 30–50% confluence. They were then transfected into cells using TurboFect™ siRNA Transfection Reagent (Fermentas, Vilnius, Lithuania) according to the manufacturer's protocol. Cells were collected after 48–72 hrs for further experiments.

### Transient transfection with miR-184 mimics and its inhibitor

miR-184 mimics and its inhibitor was designed and synthesized by Guangzhou RiboBio (RiboBio Inc). The sequences of miR-184 mimics or its inhibitor and their respective controls are shown in Table S2. Twenty-four hours prior to transfection, lung cancer cells A549 and SPAC1 were plated onto a 96-well or 6-well plates (Nest, Biotech) at 30–50% confluence. They were then transfected into cells using TurboFect™ siRNA Transfection Reagent (Fermentas) according to the manufacturer's protocol. Cells were collected after 48–72 hrs for further experiments.

### RNA isolation, reverse transcription and qRT-PCR

RNA was extracted from the lung cell lines using Trizol (Takara, Shiga, Japan). For miR-184 qRT-PCR, RNA was transcribed into cDNA and amplified with specific sense primers (Table S3), and general antisense primer was supplied by manufacturer using miRNA PrimeScript® RT Enzyme Mix kit according to the manufacturer's instructions (Ambion, Austin, TX, USA). The assays were performed in accordance with manufacturer's instructions (Takara). PCR reactions for each gene were repeated three times. The expression of miRNA was normalized to U6 (Table S3) using the 2^−ΔΔCt^ method.

### MTT assay

The rate of *in vitro* cell proliferation was assessed using 3-(4, 5-dimethylthiazol-2-yl)-2, 5-diphenyltetrazolium bromide (MTT) assay. For CCDC19 overexpression, cells were seeded in 96-well plates at a density of 1000 cells/well. The cells were incubated for 1, 2, 3, 4, 5, 6 or 7 days. Twenty microlitres of MTT (5 mg/ml; Sigma-Aldrich, St. Louis, MO, USA) was added to each well and incubated for 4 hrs. At the end of incubation, the supernatants were removed, and 150 μl of DMSO (Sigma-Aldrich) was added to each well. For siRNA-CCDC19, the cells were incubated for 1, 2 and 3 days. Twenty microlitres of MTT (5 mg/ml; Sigma-Aldrich) was added to each well and incubated for 4 hrs. At the end of incubation, the supernatants were removed, and 150 μl of DMSO (Sigma-Aldrich) was added to each well. The absorbance value (OD) of each well was measured at 490 nm. Experiments were performed three times.

### Colony formation assay

Cells were plated in 6-well culture plates at 100 cells/well. Each cell group had two wells. After incubation for 13 days at 37°C, cells were washed twice with PBS and stained with the Giemsa solution. The number of colonies containing ≥50 cells was counted under a microscope. The colony formation efficiency was calculated as: (number of colonies/number of cells inoculated) × 100%.

### Cell cycle analysis

Cells were seeded on 10-cm diameter plates in RPMI 1640 containing 10% NBCS. After incubation for 48 hrs, a total of 5 × 10^6^ cells were harvested, rinsed with cold PBS, and fixed with 70% ice-cold ethanol for 48 hrs at 4°C. Fixed cells were rinsed with cold PBS followed by incubation with PBS containing 10 μg/ml propidium iodide and 0.5 mg/ml RNase A for 30 min. at 37°C. The DNA content of labelled cells was analysed using FACS cytometry (BD Biosciences, Orlando, FL, USA). Each experiment was performed in triplicate.

### *In vivo* tumourigenesis in nude mice

A total of 1 × 10^6^ logarithmically growing A549 and SPAC1 cells transfected with pGC-FU-GFP-CCDC19 and the mock pGC-FU-GFP vector (pGC-FU-GFP-CCDC19-A549/pGC-FU-GFP-A549, *N* = 4; pGC-FU-GFP-CCDC19-SPCA1/pGC-FU-GFP-SPCA1, *N* = 5) in 0.1 ml RPMI 1640 medium were subcutaneously injected into the left flank of 4–6-week-old BALB/c nu/nu mice. The mice were maintained in a barrier facility on HEPA-filtered racks. The animals were fed an autoclaved laboratory rodent diet. All animal studies were conducted in accordance with the principles and procedures outlined in Southern Medical University Guide for the Care and Use of Animals under assurance number SCXK (Guangdong) 2008-0002. After 21 days (A549) or 23 days (SPCA1), the mice were killed and tumour tissues were excised and weighed.

### MiRNA target validation

C-Myc was predicted to be a directly regulated target of miR-184 by miRwalk software and RNAhybrid (Fig. S1). An amplified 309-bp fragment of C-Myc CDS (coding sequence) was cloned into psiCHECK-2 vector [s named wild-type (wt)] by a pair of specific primer (Table S4). Site-directed mutagenesis of the miR-184 binding sites in the C-Myc CDS was performed with GeneTailor Site-Directed Mutagenesis System (Invitrogen, Carlsbad, CA, USA) [named mutant (mt)]. For reporter assays, wt or mt vector and the control vector psiCHECK-2 vector were cotransfected into 293FT cells with miR-184 mimics or inhibitor. Luciferase activity was measured at 48 hrs after transfection using the Dual-Luciferase Reporter Assay System (Promega, Madison, WI, USA).

### Chromatin immunoprecipitation assay

The promoter region of human miR-184 (−1500 to 100 bp) was predicted to contain AP-1 (C-Jun) binding sites (Fig. S2), which suggested that C-Jun directly regulated the expression of miR-184. DNA–protein complexes were immunoprecipitated from A549 cells by using the Chromatin Immunoprecipitation Kit (Millipore, Darmstadt, Germany) according to the manufacturer's protocol with the following polyclonal antibodies: anti-C-Jun and normal mouse IgG (Millipore), the latter served as a control for non-specific DNA binding. The precipitated DNA was subjected to qPCR analysis by using specific primers. The primers (Table S5) were utilized to amplify the miR-184 promoter region.

### Luciferase reporter assays of miR-184 promoter

The miR-184 promoter sequence was constructed into pGL3 Luciferase Reporter Vector (Promega). Further, the three reporters with mutations in the putative C-Jun binding sites of the miR-184 promoter (MT1, MT2 and MT1+2; Fig. S2) were prepared using GeneTailor Site-Directed Mutagenesis System (Invitrogen). The reporter constructs were respectively transfected into 293FT cells with expression vectors pcDNA3.1 encoding C-Jun. Twenty-four hours after transfection, cell lysates were prepared and luciferase activity was determined using Luciferase Assay System with Reporter Lysis Buffer (Promega) according to the manufacturer's instructions.

### Statistical analysis

All data were analysed for statistical significance using SPSS 13.0 software. The chi-squared test was applied to examine the relationship between CCDC19 expression levels and clinicopathological characteristics. Survival analysis was performed with Kaplan–Meier method. Multivariate Cox proportional hazards method was used for analysing the relationship between the variables and patient's survival time. Two-tailed Student's *t*-test was used for comparisons of two independent groups. One-way anova was used to determine the differences between groups for all *in vitro* analyses. A *P* value of less than 0.05 was considered statistically significant.

## Results

### Down-regulated protein expression of CCDC19 is unfavourable for NSCLC prognosis

**Fig. 1:**
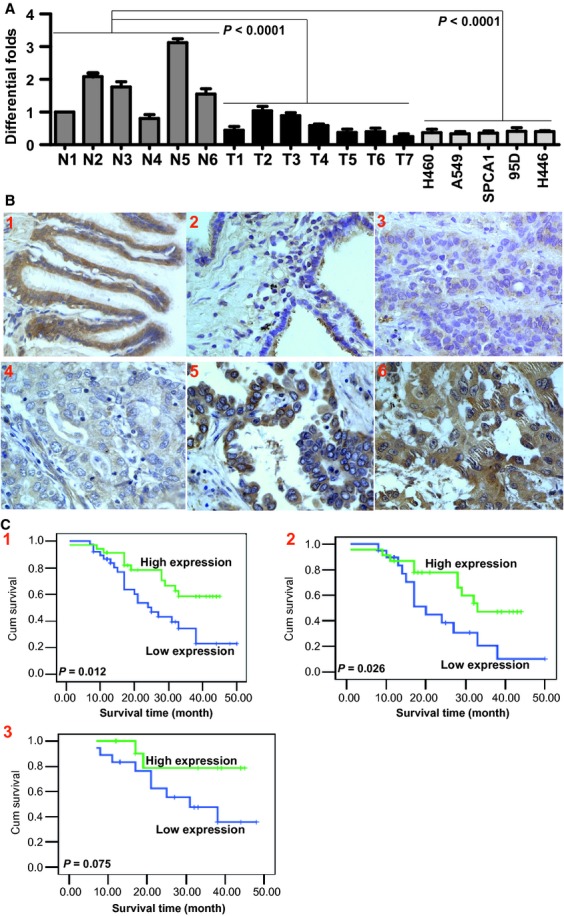
Down-regulation of CCDC19 expression is unfavourable for NSCLC prognosis. (**A**) Compared with normal lung tissues, CCDC19 mRNA expression was markedly decreased in NSCLC tissues (*P* < 0.001) and cells (*P* < 0.001) by qPCR examination. N: normal tissues; T: tumour tissues; (**B**) Compared with lung tissues, CCDC19 protein expression was significantly down-regulated in nasopharyngeal carcinoma (NPC) tissues. 1 and 2: High expression of CCDC19 in lung tissues; 3 and 4: Low expression of CCDC19 in NSCLC tissues; 5 and 6: High expression of CCDC19 in NSCLC tissues; (**C**) Decreased expression of CCDC19 shortened the survival time of NSCLC patients. 1: Lower levels of CCDC19 reduced the survival time of NSCLC patients; 2: Down-regulation of CCDC19 expression significantly reduced the survival time of adenocarcinoma patients; 3: Decreased CCDC19 expression reduced the survival time of squamous cell carcinoma patients.

Markedly reduced expression of CCDC19 mRNA was observed in lung cancer cell lines and tissues compared to normal lung samples (Fig. 1A). Protein expression levels of CCDC19 were then measured in samples of 73 archived paraffin-embedded lung cancer and 22 paracancerous lung tissues using immunohistochemical staining (Fig. 1B1-6). CCDC19 protein was highly expressed in bronchus epithelium of lung tissues compared to lung cancer samples (*P* = 0.038; Table S6). Further, we analysed the relationship between clinicopathological characteristics and CCDC19 expression levels in individuals with NSCLC (Table S7). We did not find a significant association between CCDC19 expression levels and patient's age, sex, smoking status, family tumour history, location, tumour size (T classification), lymph node metastasis (N classification), distant metastasis (N classification) or clinical stage (I-II *versus* III-IV) in 73 NSCLC patients.

### Survival analysis

To investigate the prognostic value of CCDC19 expression for lung cancer, we assessed the association between protein levels and patients' survival using Kaplan–Meier analysis with the log-rank test. We observed that the level of CCDC19 protein expression was significantly correlated with overall survival time, as patients with lower levels of CCDC19 expression had poorer survival (Fig. 1C) than those with greater CCDC19 expression (*P* = 0.012) in NSCLC or squamous cell carcinoma (*P* = 0.075), or adenocarcinoma (*P* = 0.026). Univariate analysis showed that only CCDC19 expression was significantly correlated with patients' survival time (*P* = 0.016). Multivariate analysis of the levels of CCDC19 protein expression adjusted for all other NSCLC patient factors determined that CCDC19 expression was an independent prognostic factor for NSCLC (*P* = 0.003; Table S8).

### CCDC19 inhibits cell proliferation and cell cycle transition in NSCLC

**Fig. 2:**
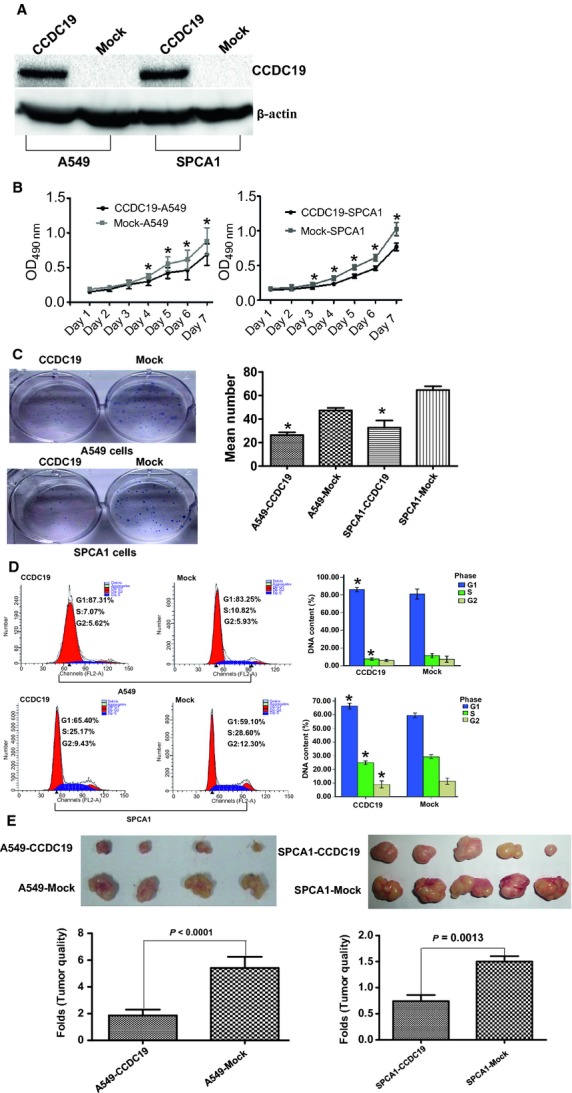
Overexpression of CCDC19 suppresses cell proliferation and cell cycle transition *in vitro* and *in vivo* NSCLC. (**A**) Using lentiviral-mediated transfer of the CCDC19 gene, restoration of CCDC19 protein expression was observed in A549 and SPCA1 cells compared to their respective mock cells by Western blot. Because of the fusion protein from CCDC19 and green fluorescent protein (GFP), the fusion protein of their respective mock cells cannot be detected. (**B** and **C**) *In vitro* proliferative ability of NSCLC cells was significantly suppressed in CCDC19-overexpressing A549 and SPCA1 cells compared to their respective mock cells by MTT and plate clone formation assays. (**D**) Restored CCDC19 expression markedly inhibited cell cycle transition from G1 to S and G2 *in vitro*. (**E**) Compared with respective mock cells, tumourigenicity of A549 and SPCA1 cells with CCDC19 overexpression was markedly reduced *in vivo*. Data were presented as mean ± SD for three independent experiments (*P < 0.05).

To study its biological functions, we ectopically expressed CCDC19 in lung cell lines A549 and SPAC1 using lentivirus. Western blotting assay showed that CCDC19-GFP fusion protein expression in A549 and SPAC1 cells was markedly increased compared with their basal levels in respective empty vector control cells (Fig. 2A). Next, we studied the rate of cell proliferation of CCDC19-expressing A549 and SPAC1 cells. The growth curves determined by MTT assay showed that CCDC19 significantly suppressed cell proliferation of these two cell lines (Fig. 2B). CCDC19-expressing A549 and SPAC1 cells also formed significantly less colonies than those of their respective mock cells in a colony formation assay (*P* = 0.003 for A549; *P* = 0.01 for SPCA1; Fig. 2C), suggesting the inhibitory effect of CCDC19 on anchorage-dependent growth of NSCLC cells. Furthermore, we measured cell cycle distribution in CCDC19-expressing A549 and SPAC1 cells and observed that the G1 phase population was markedly increased, while the S phase population was significantly decreased compared to their respective mock cells (Fig. 2D). Finally, we confirmed the growth suppressive effect of CCDC19 *in vivo* with a tumourigenesis study by inoculating CCDC19-expressing A549 and SPAC1 cells into nude mice (Fig. 2E). The average weights of these two polyclonal cell lines were significantly decreased compared with their respective mock cells. Our results suggested a significant inhibitory effect of CCDC19 on tumourigenesis *in vivo*.

### Knocking down CCDC19 expression increases cell proliferation of NSCLC cells *in vitro*

**Fig. 3:**
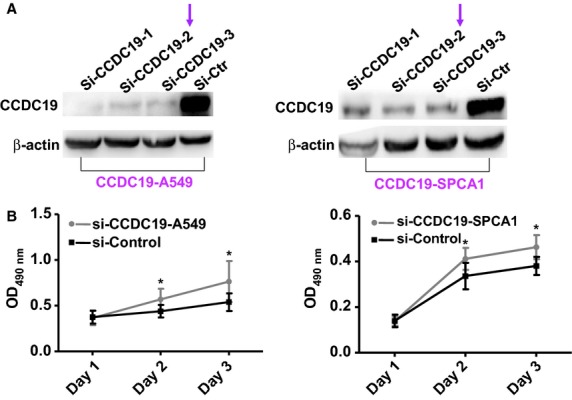
Knockdown of CCDC19 expression stimulates cell proliferation in NSCLC. (**A**) Specific siRNAs were used to suppress the expression of CCDC19 in CCDC19-overexpressed A549 and SPCA1 cells by Western blot examination. (**B**) Cell growth ability was significantly enhanced in siCCDC19-treated A549 and SPCA1 cells.

To further confirm the biological function of suppressed CCDC19 in NSCLC, we used a siRNA to specifically knock down CCDC19 expression in CCDC19-overexpressed A549 and SPAC1 cells. Decreased expression of CCDC19 protein was confirmed by Western blotting in these two cells compared to their respective cells with si-Ctr (Fig. 3A). Using an MTT assay, we found that si-CCDC19 A549 and SPAC1 cells had an elevated growth rate over a 3-day period (*P* < 0.001; Fig. 3B).

### CCDC19 modulates the expression of PI3K/AKT and its downstream signals including cell cycle pathway and C-Jun in NSCLC

**Fig. 4:**
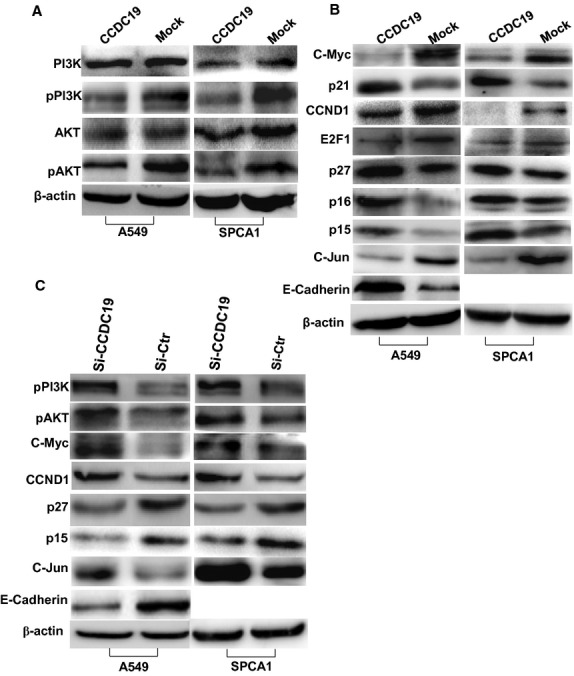
CCDC19 controls the expression of cell cycle signals and transcription factor C-JUN in NSCLC *via* PI3K/AKT pathway. (**A**) Overexpressed CCDC19 suppressed the expression of pPI3K and p-AKT, but not their total protein levels in CCDC19-overexpressing A549 and SPCA1 cells compared to their respective mock cells. (**B**) Restored CCDC19 inhibited the expression of C-Myc, CCND1, E2F1 and C-JUN and induced the expression of p21, p27, p15 and p16 in A549 and SPCA1 cells. Increased expression of E-Cadhein was only observed in CCDC19-overexpressed A549 cells. SPCA1 cells did not express E-Cadherin. (**C**) Knocking down CCDC19 by siRNA restored the activation of pPI3K and pAKT, stimulated the expression of C-Myc and CCND1, as well as repressed p15 and p21 expression in CCDC19-overexpressed A549 and SPCA1 cells. Reduced expression of E-Cadherin was only observed in CCDC19-suppressed A549 cells. SPCA1 cells did not express E-Cadherin.

PI3K/AKT is a classically oncogenic signal and its activation participates in the pathogenesis of tumours [18–20]. We first examined the effects of CCDC19 on PI3K/AKT in NSCLC. Western blot analysis showed that overexpression of CCDC19 significantly reduced the expression of phos-PI3K and phos-AKT respectively, but not their total protein levels (Fig. 4A). Modulation of PI3K/AKT pathway affects its downstream signals including cell cycle factors and transcription factor C-Jun, thus we examined their protein expression in CCDC19-overexpressing lung cancer A549 and SPCA1 cells. Ectopic CCDC19 blocked the expression of C-Myc, CCND1, E2F1 and C-Jun, and induced the expression of p21, p15, p16 and p27 (Fig. 4B). Conversely, knocking down exogenous CCDC19 expression stimulated the expression of C-Myc, CCND1, E2F1 and C-Jun, and suppressed the expression of p21, p15 and p27 in A549 and SPCA1 cells with CCDC19 overexpression (Fig. 4C). Attributing to the fact that SPCA1 cells did not express E-cadherin, increased or decreased expression of E-Cadherin was only respectively observed in CCDC19-overexpressed or CCDC19-suppressed A549 cells (Fig. 4B and C).

### CCDC19 positively modulates the expression of miR-184 and miR-206

**Fig. 5:**
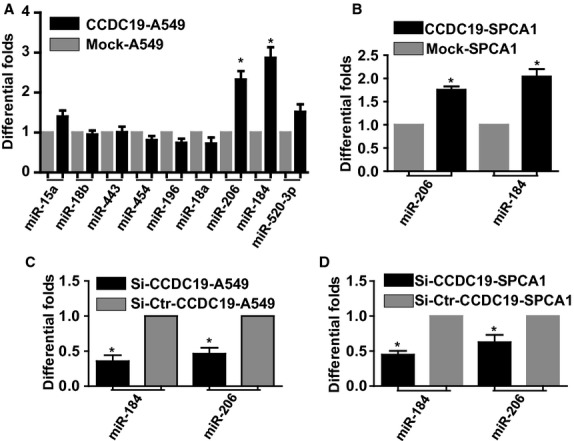
CCDC19 positively regulates the expression of miR-184 in NSCLC. (**A**) miR-184 and miR-206 were notably up-regulated in CCDC19-overexpressing A549 cells by miRNA qPCR analysis. (**B**) qPCR confirmed the elevated expression of miR-184 and miR-206 in CCDC19-overexpressing SPCA1 cells. (**C** and **D**) Knocking down CCDC19 suppressed the expression of miR-184 and miR-206 in CCDC19-overexpressing A549 and SPCA1 cells.

To investigate the effect of CCDC19 on miRNAs, we examined the expression of miRNAs in A549-overexpressed cells by qPCR (Fig. 4A). Expression of miR-184 and miR-206 was markedly induced in CCDC19-overexpressing A549 cells (Fig. 5A). Interestingly, a similar result was also observed in CCDC19-overexpressing SPCA1 cells (Fig. 5B). We observed an inverse change in miR-184 and miR-206 after knocking down ectopic CCDC19 expression in A549 and SPCA1 NSCLC cells (Fig. 5C and D). The fold change of miR-184 was more than twofold in each of these experiments.

### miR-184 suppresses cell proliferation by modulating cell cycle transition signals

**Fig. 6:**
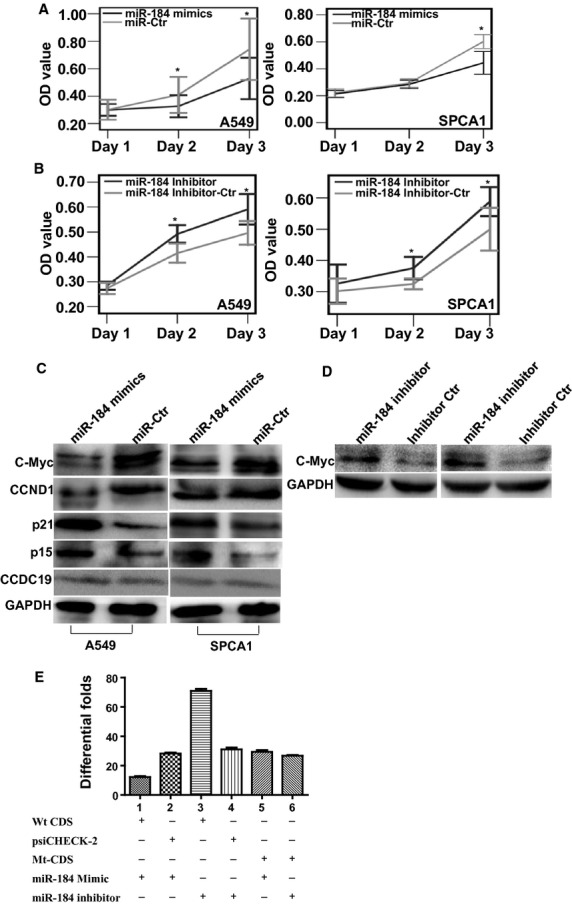
miR-184 directly targets C-MYC-mediated cell cycle signal suppressing cell growth in NSCLC. (**A**) The introduction of miR-184 mimics suppressed cell growth in lung cancer A549 and SPCA1 cells by MTT assay. (**B**) Transfection of miR-184 inhibitor induced cell proliferation in A549 and SPCA1 lung cancer cells by MTT assay. (**C**) The introduction of miR-184 mimics suppressed the expression of C-Myc and CCND1 and stimulated p21 and p15 expression in A549 and SPCA1 cells. (**D**) miR-184 inhibitor suppressed the expression C-Myc in A549 and SPCA1 cells. (**E**) Luciferase reporter assay was used to determine miR-184 direct targeting of the C-Myc coding region. Data are presented as mean ± SD for three independent experiments (*P < 0.05).

To examine the effect of miR-184 on cell proliferation, miR-184 mimics and its inhibitor were respectively transfected into lung cancer cell lines according to the manufacturer's instructions. Compared with their respective controls, miR-184 mimics suppressed cell growth (Fig. 6A), while its inhibitor with respective suppression efficiency of 78.14% and 72.38% for miR-184 (Fig. S3) in A549 and SPCA1 cells (Fig. 6B) induced cell proliferation by MTT assays. Further, we investigated the molecular basis of miR-184 cell proliferation inhibition in lung cancer. Interestingly, similar to CCDC19-mediated cell cycle pathway, miR-184 mimics suppressed the expression of C-Myc and CCND1 while inducing p21 and p15 expression (Fig. 6C).

### miR-184 directly targeted oncogenic transcription factor C-Myc

To investigate the molecular basis of miR-184 cell cycle regulation in lung cancer, we analysed the modulated targets of miR-184 by miRwalk assay and RNAhybrid. Oncogenic transcription factor C-Myc, an upstream regulator of cell cycle [21,22], was found to be its potentially direct targets. Transfection of miR-184 mimics down-regulated the protein expression of C-Myc in lung cancer cell lines A549 and SPCA1. Subsequently, we transfected miR-184 inhibitor into these two cells and observed elevated expression of C-Myc (Fig. 6D). We further performed a luciferase reporter assay to determine whether miR-184 could directly target the C-Myc coding region (CDS) in 293FT cells. miR-184 mimics induced a significant decrease of luciferase activity of wt vector C-Myc CDS (Fig. 6E, lanes 1 and 2; *P* < 0.001) or an obvious increase of luciferase activity by transfecting miR-184 inhibitor (Fig. 6E, lanes 3 and 4; *P* < 0.001) compared with miR controls. The activity of mt vectors was unaffected (Fig. 6E, lanes 5 and 6; *P* = 0.36) by miR-184 mimics or inhibitor, consistent with our previous findings. Taken together, these results strongly supported that C-Myc is a direct target of miR-184.

### C-Jun directly negatively modulates the expression of miR-184

**Fig. 7:**
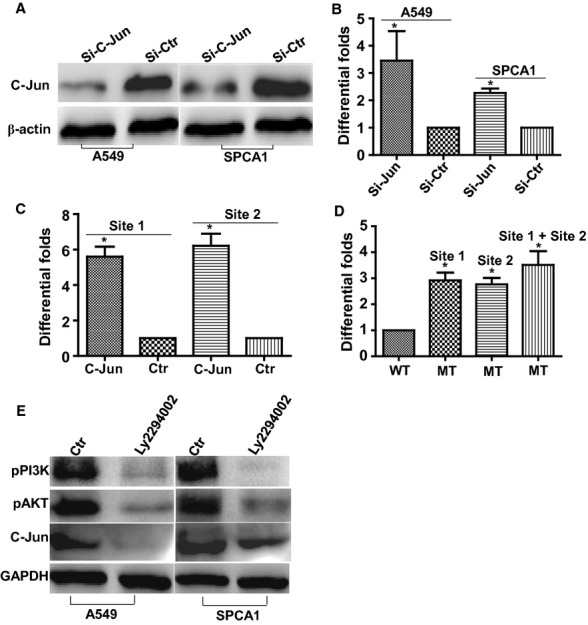
PI3K/AKT signalling modulates C-Jun-mediated direct suppression of miR-184 in NSCLC. (**A**) siRNA was used to suppress C-Jun expression by Western blot. (**B**) Inhibition of C-Jun expression by siRNA stimulated the expression of miR-184 in A549 and SPCA1 cells. (**C**) Cross-linked chromatin preparation from mock and pcDNA3.1-C-Jun-transfected A549 cells was immunoprecipitated with anti-C-Jun or a normal rabbit IgG. The C-Jun binding sites on the immunoprecipitated DNA were determined by quantitative RT-PCR. Amplification of input chromatin (input) prior to immunoprecipitation was served as a positive control for chromatin extraction and PCR amplification. Chromatin immunoprecipitation using a non-specific antibody (normal human IgG) served as negative controls. qPCR analysis indicated that C-Jun could bind more DNA in Site1 and Site2 of the miR-184 promoter region in pcDNA3.1-C-Jun-transfected A549 cells than that in control pcDNA3.1-transfected A549 cells. (**D**) The reporter constructs of Mutants (MT1, MT2, MT1+2) and normal control were respectively transfected into 293FT cells with expression vectors pcDNA3.1 encoding C-Jun and luciferase activity of miR-184 promoter was significantly increased in these mutant constructs compared to normal control. (**E**) Ly294002, a specific inhibitor (50 nM) of pPI3K, was used to suppress the expression of PI3K reduced the expression pPI3K, p-AKT and C-JUN in A549 and SPCA1 cells. Data were presented as mean ± SD for three independent experiments (*P < 0.05).

To confirm that miR-184 is directly regulated by C-Jun, we first used siRNA [23] to suppress the expression of C-Jun in lung cancer A549 and SPCA1 cell lines as confirmed by Western blot (Fig. 7A).The expression of miR-184 was markedly increased by qPCR analysis in both cell lines (Fig. 7B). Subsequently we used chromatin immunoprecipitation combined with qPCR analysis to confirm that C-Jun could bind to the putative miR-184 promoter (Fig. 7C). Using the C-Jun binding site mutants, we found that luciferase activity of miR-184 promoter was significantly increased in 293FT cells (Fig. 7D). Finally, we used a specific inhibitor of PI3K to suppress the expression of PI3K and observed that the protein expression of C-Jun was decreased in lung cancer cell lines A549 and SPCA1 (Fig. 7E). These results demonstrated that CCDC19 stimulated the expression of miR-184 through PI3K/AKT/C-Jun pathway.

## Discussion

In a recent investigation, we updated the CCDC19 ORF sequence and investigated its role in NPC cells. Our results preliminarily suggested CCDC19 as a tumour suppressor in NPC [7,8]. In this study, we observed that the expression of CCDC19 mRNA and protein was significantly reduced in NSCLC tissues and cells compared to normal lung tissues by qPCR and Western blot. Further, we found that protein expression of CCDC19 was decreased in NSCLC tissues compared to normal tissues by immunohistochemistry. These results were not only consistent with our previous investigation in NPC [7,8], but also hinted that the loss of CCDC19 expression was involved in the stages of initiation and pre-cancerous lesion of NSCLC.

We previously observed that decreased protein expression of CCDC19 was unfavourable for clinical progression and poor prognosis of NPC [7,8]. In this investigation, we found that although reduced expression of CCDC19 was not associated with clinical features, it was significantly correlated with the prognosis of NSCLC. Furthermore, a positive correlation was observed between down-regulation of CCDC19 and lung adenocarcinoma or squamous cell lung cancer patients by strata analysis against NSCLC. Patients with lower levels of CCDC19 expression had poorer survival time than those with greater CCDC19 expression. Furthermore, we observed that reduced CCDC19 expression was an independent prognostic factor for NSCLC patients in this study. These studies consistently demonstrated that down-regulated CCDC19 levels play an unfavourable role in NSCLC pathogenesis, a correlation which has not been previously reported.

In a prior study, CCDC19 had been described to suppress cell proliferation, migration, invasion and cell cycle progression in squamous cell carcinoma of nasopharynx [7,8]. In this study, we examined the biological functions of CCDC19 in NSCLC. Similar to our previous reports, CCDC19 not only significantly inhibited the proliferation and G1/S cell cycle transition of NSCLC cells *in vitro* but also markedly suppressed tumourigenicity in an *in vivo* xenograft animal model. Our studies strongly suggest a suppressive role of CCDC19 in the development of NSCLC.

In previous studies, CCDC19 had been shown to block cell growth and cell cycle transition by inducing p21 expression and decreasing CCNA1 expression in NPC [7]. CCDC19-mediated growth suppression and cell cycle arrest was mainly attributed to reduce the expression of C-Jun and positive cell cycle regulators including C-Myc, CCND1 and E2F1 [24,25] as well as increased the expression of negative cell cycle regulators including p21, p27, p15 and p16 [26–29]. Further, we observed that PI3K/AKT, an upstream pathway modulating C-Jun and cell cycle transition, was significantly inactivated in NSCLC cells. These results demonstrated that CCDC19 suppressed cell proliferation and cell cycle transition by inactivating PI3K/AKT-mediated modulation of the cell cycle and C-Jun. Furthermore, CCDC19-mediated inhibition of cell proliferation, migration and invasion was possibly correlated with down-regulation of E2F1, which suppressed the expression of cell division cycle 6 (CDC6), a protein regulating DNA replication, activating and maintaining the checkpoint mechanisms in the cell cycle, and further induced the expression of cell cycle inhibitor p16 and epithelial to mesenchymal transition (EMT) marker E-Cadherin in NSCLC [29,30]. However, this speculation is still to be determined.

miRNAs are a class of 19- to 30-nucleotide-long, non-coding RNAs widely expressed in eukaryotes and predominantly inhibit gene expression at the post-transcriptional level and have been displayed to have links with various types of cancer [31–34]. However, their mediated tumour growth suppression of CCDC19 in tumours has not been reported. In this study, we used qPCR to examine differential miRNA expression and discovered that miR-184 and miR-206 were significantly up-regulated in CCDC19-overexpressing A549 and SPCA1 lung cancer cells compared to their respective controls. In a previous report, miR-206, but not miR-184, was been found to act as a tumour suppressor to inhibit cell growth, invasion and metastasis in lung cancer [35]. In this study, we investigated the role of miR-184 in NSCLC. Dysregulation of miR-184 was reported to be a potential oncomir promoting cell proliferation and blocking cell apoptosis in tongue and hepatocellular carcinoma [11,12]. Subsequently, miR-184 was found to be involved in suppressing cell survival and growth by targeting AKT2 in neuroblastoma cells [14]. In our study, miR-184 was found to suppress cell proliferation by inhibiting oncogenic cell cycle factors C-Myc and CCND1, and inducing the expression of cell cycle suppressors p15 and p21 in NSCLC, similar to the role of CCDC19. However, miR-184 did not modulate the expression of endogenous CCDC19, which suggested it as a downstream factor modulated by CCDC19 in NSCLC cells (Fig. S4).

To understand the molecular mechanism of CCDC19-modulated suppression of cell growth in NSCLC, we observed that C-Myc, a key initial factor which suppresses p15 and p21 expression and stimulates CCND1 expression which promotes G1 to S transition [21], was a direct target of miR-184. Further, we found that C-Jun (AP1), an oncogenic transcription factor regulated by CCDC19, directly bound to the promoter of miR-184 and suppressed its expression in NSCLC. Furthermore, we observed that PI3K/AKT pathway, a classical signal pathway which promotes tumour pathogenesis [18,19], was inactivated by CCDC19 in NSCLC cells and positively regulated C-Jun expression. Our results demonstrated that miR-184 direct targeting of C-Myc was responsible for the CCDC19-mediated suppression of cell growth through the PI3K/AKT/C-Jun pathway.

In summary, we observed that decreased CCDC19 cytoplasmic expression facilitated poor prognosis of NSCLC. We provided evidence that CCDC19 suppresses cell growth by inactivating CCDC19-mediated suppression of growth involving the direct interaction of miR-184 with C-Myc, and transcription suppression of miR-184 by CCDC19-inactivated PI3K/AKT/C-Jun pathway (Fig. S5).

## Supporting information

**Figure S1** C-Myc was predicted to be a potential target of miR-184 by RNAhybrid software.

**Figure S2** C-Jun binding sites and its mutant sites in miR-184 promoter. MT1: The mutation of site 1; MT2: The mutation of site 2; MT1+2: The combined mutations of MT1 and MT2.

**Figure S3** The expression of miR-184 was suppressed by its specific inhibitor in A549 and SPCA1 cells.

**Figure S4** miR-184 did not regulate the expression of CCDC19 in NSCLC.

**Figure S5** Ideograph of tumour suppressor CCDC19 in modulating cell proliferation.

**Table S1** siRNAs for CCDC19.

**Table S2** Sequences of miR-184 mimics and negative control, miR-184 inhibitor and inhibitor negative control.

**Table S3** Sequences of miR-184 and U6.

**Table S4** Primer sequences for C-MYC CDS.

**Table S5** ChIP Primer sequences for miR-184 promoter.

**Table S6** Down-regulation of NESG1 protein in NPC compared to NP epithelium tissues.

**Table S7** Correlation between the clinicopathological characteristics and expression of NESG1 protein in lung cancer.

**Table S8** Summary of univariate and multivariate Cox regression analysis of overall survival duration.

## References

[1] Ferlay J, Shin HR, Bray F (2010). Estimates of worldwide burden of cancer in 2008: GLOBOCAN 2008. Int J Cancer.

[2] Hanai JI, Doro N, Seth P (2013). ATP citrate lyase knockdown impacts cancer stem cells *in vitro*. Cell Death Dis.

[3] Kim HR, Kim DJ, Kang DR (2013). Fibroblast growth factor receptor 1 gene amplification is associated with poor survival and cigarette smoking dosage in patients with resected squamous cell lung cancer. J Clin Oncol.

[4] Sagne C, Marcel V, Amadou A (2013). A meta-analysis of cancer risk associated with the TP53 intron 3 duplication polymorphism (rs17878362): geographic and tumor-specific effects. Cell Death Dis.

[5] Zabarovsky ER, Lerman MI, Minna JD (2002). Tumor suppressor genes on chromosome 3p involved in the pathogenesis of lung and other cancers. Oncogene.

[6] Li Z, Yao K, Cao Y (1999). Molecular cloning of a novel tissue-specific gene from human nasopharyngeal epithelium. Gene.

[7] Liu Z, Li X, He X (2011). Decreased expression of updated NESG1 in nasopharyngeal carcinoma: its potential role and preliminarily functional mechanism. Int J Cancer.

[8] Liu Z, Luo W, Zhou Y (2011). Potential tumor suppressor NESG1 as an unfavorable prognosis factor in nasopharyngeal carcinoma. PLoS ONE.

[9] Aboobaker AA, Tomancak P, Patel N (2005). Drosophila microRNAs exhibit diverse spatial expression patterns during embryonic development. Proc Natl Acad Sci USA.

[10] Weitzel RP, Lesniewski ML, Greco NJ (2011). Reduced methyl-CpG protein binding contributing to miR-184 expression in umbilical cord blood CD4+ T-cells. Leukemia.

[11] Gao B, Gao K, Li L (2014). miR-184 functions as an oncogenic regulator in hepatocellular carcinoma (HCC). Biomed Pharmacother.

[12] Wong TS, Liu XB, Wong BY (2008). Mature miR-184 as potential oncogenic microRNA of squamous cell carcinoma of tongue. Clin Cancer Res.

[13] Leng HM, Qian WP, Zhou L (2011). Abnormal expression and significance of MIR-184 in human renal carcinoma. Beijing Da Xue Xue Bao.

[14] Foley NH, Bray IM, Tivnan A (2010). MicroRNA-184 inhibits neuroblastoma cell survival through targeting the serine/threonine kinase AKT2. Mol Cancer.

[15] Fang ZH, Dong CL, Chen Z (2009). Transcriptional regulation of survivin by c-Myc in BCR/ABL-transformed cells: implications in anti-leukaemic strategy. J Cell Mol Med.

[16] Popescu NC, Zimonjic DB (2002). Chromosome-mediated alterations of the MYC gene in human cancer. J Cell Mol Med.

[17] Edge SB, Compton CC (2010). The American Joint Committee on Cancer: the 7th edition of the AJCC cancer staging manual and the future of TNM. Ann Surg Oncol.

[18] Wang Y, Wang W, Wang L (2012). Regulatory mechanisms of interleukin-8 production induced by tumour necrosis factor-α in human hepatocellular carcinoma cells. J Cell Mol Med.

[19] Chang L, Graham PH, Hao J (2013). Acquisition of epithelial-mesenchymal transition and cancer stem cell phenotypes is associated with activation of the PI3K/Akt/mTOR pathway in prostate cancer radioresistance. Cell Death Dis.

[20] Yi YW, Hong W, Kang HJ (2013). Inhibition of the PI3K/AKT pathway potentiates cytotoxicity of EGFR kinase inhibitors in triple-negative breast cancer cells. J Cell Mol Med.

[21] Steiner P, Rudolph B, Müller D (1996). The functions of Myc in cell cycle progression and apoptosis. Prog Cell Cycle Res.

[22] Nathalie HV, Chris P, Serge G (2009). High kallikrein-related peptidase 6 in non-small cell lung cancer cells: an indicator of tumour proliferation and poor prognosis. J Cell Mol Med.

[23] Yu X, Zhen Y, Yang H (2013). Loss of connective tissue growth factor as an unfavorable prognosis factor activates miR-18b by PI3K/AKT/C-Jun and C-Myc and promotes cell growth in nasopharyngeal carcinoma. Cell Death Dis.

[24] Walluscheck D, Poehlmann A, Hartig R (2013). ATF2 knockdown reinforces oxidative stress-induced apoptosis in TE7 cancer cells. J Cell Mol Med.

[25] Simile MM, De Miglio MR, Muroni MR (2004). Down-regulation of c-myc and Cyclin D1 genes by antisense oligodeoxy nucleotides inhibits the expression of E2F1 and *in vitro* growth of HepG2 and Morris 5123 liver cancer cells. Carcinogenesis.

[26] Lv C, Hong Y, Miao L (2013). Wentilactone A as a novel potential antitumor agent induces apoptosis and G2/M arrest of human lung carcinoma cells, and is mediated by HRas-GTP accumulation to excessively activate the Ras/Raf/ERK/p53-p21 pathway. Cell Death Dis.

[27] Wei M, Wang Z, Yao H (2011). P27(Kip1), regulated by glycogen synthase kinase-3β, results in HMBA-induced differentiation of human gastric cancer cells. BMC Cancer.

[28] Hayslip J, Montero A (2006). Tumor suppressor gene methylation in follicular lymphoma: a comprehensive review. Mol Cancer.

[29] Gonzalez S, Klatt P, Delgado S (2006). Oncogenic activity of Cdc6 through repression of the INK4/ARF locus. Nature.

[30] Sideridou M, Zakopoulou R, Evangelou K (2011). Cdc6 expression represses E-cadherin transcription and activates adjacent replication origins. J Cell Biol.

[31] Huang X, Huang F, Yang D (2012). Expression of microRNA-122 contributes to apoptosis in H9C2 myocytes. J Cell Mol Med.

[32] Yu N, Huangyang P, Yang X (2013). microRNA-7 suppresses the invasive potential of breast cancer cells and sensitizes cells to DNA damages by targeting histone methyltransferase SET8. J Biol Chem.

[33] Dai DW, Lu Q, Wang LX (2013). Decreased miR-106a inhibits glioma cell glucose uptake and proliferation by targeting SLC2A3 in GBM. BMC Cancer.

[34] Liu X, Chen Q, Yan J (2013). MiRNA-296-3p-ICAM-1 axis promotes metastasis of prostate cancer by possible enhancing survival of natural killer cell-resistant circulating tumour cells. Cell Death Dis.

[35] Wang X, Ling C, Bai Y (2011). MicroRNA-206 is associated with invasion and metastasis of lung cancer. Anat Rec.

