# Supplementary figures and images for: Candidate tumour suppressor CCDC19 regulates miR-184 direct targeting of C-Myc thereby suppressing cell growth in non-small cell lung cancers
Source: J Cell Mol Med. 2014 Jun 26;18(8):1667–79. doi: 10.1111/jcmm.12317 (PMC4190912; doi:10.1111/jcmm.12317)

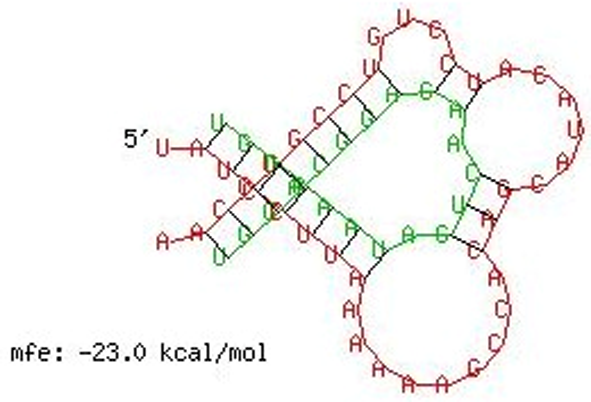

Supplement: Supplementary file 1 — Figure S1 C-Myc was predicted to be a potential target of miR-184 by RNAhybrid software. [file jcmm0018-1667-SD1.tif]

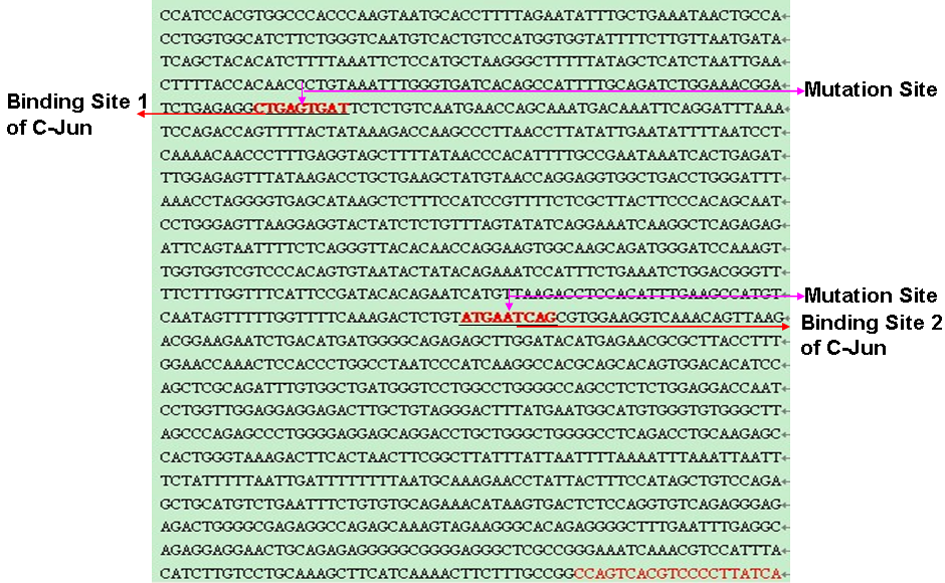

Supplement: Supplementary file 2 — Figure S2 C-Jun binding sites and its mutant sites in miR-184 promoter. MT1: The mutation of site 1; MT2: The mutation of site 2; MT1+2: The combined mutations of MT1 and MT2. [file jcmm0018-1667-SD2.tif]

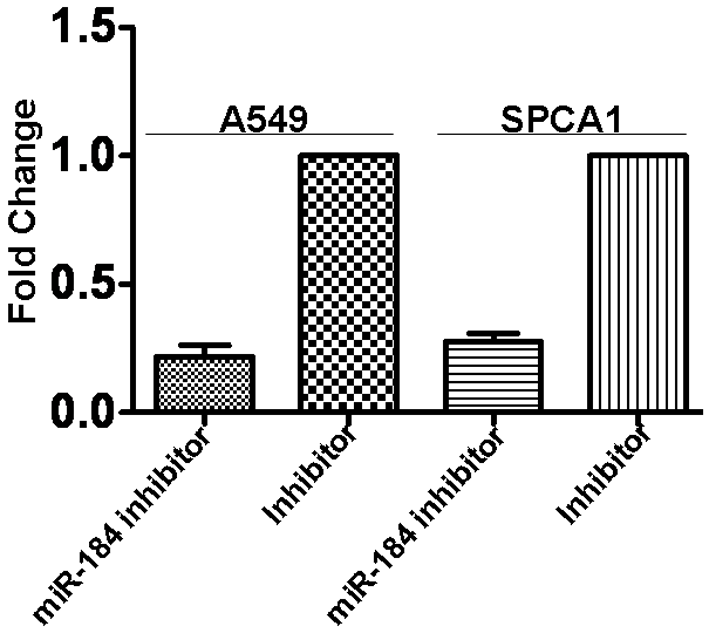

Supplement: Supplementary file 3 — Figure S3 The expression of miR-184 was suppressed by its specific inhibitor in A549 and SPCA1 cells. [file jcmm0018-1667-SD3.tif]

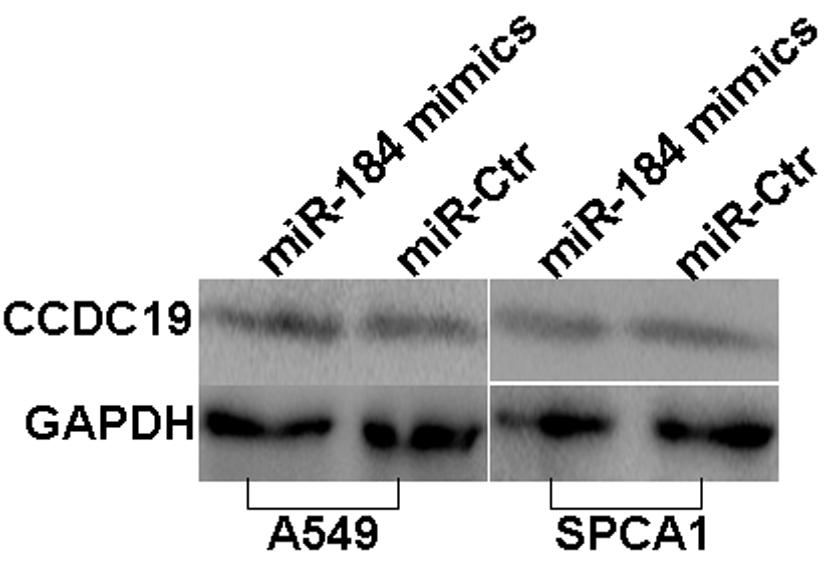

Supplement: Supplementary file 4 — Figure S4 miR-184 did not regulate the expression of CCDC19 in NSCLC. [file jcmm0018-1667-SD4.tif]

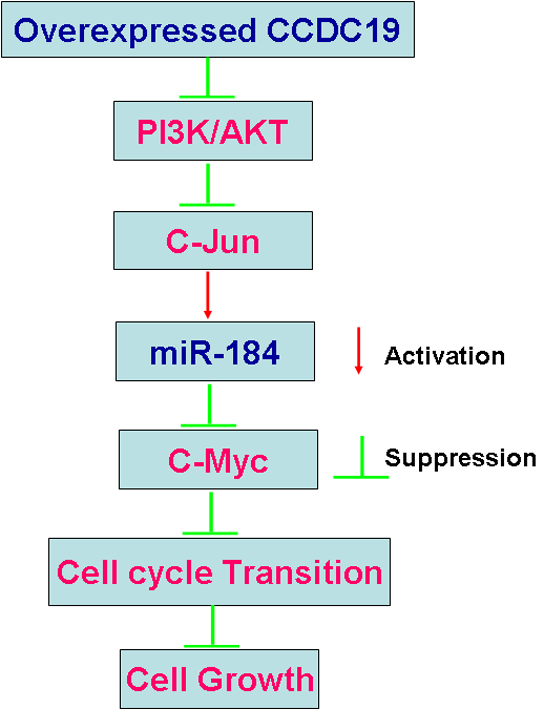

Supplement: Supplementary file 5 — Figure S5 Ideograph of tumour suppressor CCDC19 in modulating cell proliferation. [file jcmm0018-1667-SD5.tif]
