# Supplementary material for: Candidate tumour suppressor CCDC19 regulates miR-184 direct targeting of C-Myc thereby suppressing cell growth in non-small cell lung cancers
Source: J Cell Mol Med. 2014 Jun 26;18(8):1667–79. doi: 10.1111/jcmm.12317 (PMC4190912; doi:10.1111/jcmm.12317)
Supplement: Supplementary file 6 — Table S1 siRNAs for CCDC19. [file jcmm0018-1667-SD6.doc]

Table S1: siRNAs for CCDC19

| CCDC19 | Sequence |
| --- | --- |
| Si-CCDC19-Sense-1 | 5’- GAACGAAGGCAGCAACAAA dTdT -3’ |
| Si-CCDC19-anti-Sense-1 | 5’- dTdT CUUGCUUCCGUCGUUGUUU -3’ |
| Si-CCDC19-Sense-2 | 5’- GAAGAUGGCUCGAGAAGCA dTdT -3’ |
| Si-CCDC19-anti-Sense-2 | 5’- dTdT CUUCUACCGAGCUCUUCGU -3’ |
| Si-CCDC19-Sense-3 | 5’- GAGAACAGAUUGAGAAGGA dTdT -3’ |
| Si-CCDC19-anti-Sense-3 | 5’- dTdT CUCUUGUCUAACUCUUCCU -3’ |
