# Supplementary material for: Candidate tumour suppressor CCDC19 regulates miR-184 direct targeting of C-Myc thereby suppressing cell growth in non-small cell lung cancers
Source: J Cell Mol Med. 2014 Jun 26;18(8):1667–79. doi: 10.1111/jcmm.12317 (PMC4190912; doi:10.1111/jcmm.12317)
Supplement: Supplementary file 7 — Table S2 Sequences of miR-184 mimics and negative control, miR-184 inhibitor and inhibitor negative control. [file jcmm0018-1667-SD7.doc]

Table S2 Sequences of miR-184 mimics and negative control, miR-184 inhibitor and inhibitor negative control

| Gene |  | Sequence |
| --- | --- | --- |
| miR-184 mimics | Sense | 5’ UGGACGGAGAACUGAUAAGGGU3’ |
|  | Antisense | 5’ CCUUAUCAGUUCUCCGUCCAUU3’ |
| Negative control | Sense | 5’ UUCUCCGAACGUGUCACGUTT3’ |
|  | Antisense | 5’ ACGUGACACGUUCGGAGAATT3’ |
| miR-184 inhibitor |  | 5’ ACCCUUAUCAGUUCUCCGUCCA3’ |
| Inhibitor negative control |  | 5’ CAGUACUUUUGUGUAGUACAA3’ |
