# Supplementary material for: Candidate tumour suppressor CCDC19 regulates miR-184 direct targeting of C-Myc thereby suppressing cell growth in non-small cell lung cancers
Source: J Cell Mol Med. 2014 Jun 26;18(8):1667–79. doi: 10.1111/jcmm.12317 (PMC4190912; doi:10.1111/jcmm.12317)
Supplement: Supplementary file 8 — Table S3 Sequences of miR-184 and U6. [file jcmm0018-1667-SD8.doc]

Table S3 Sequences of miR-184 and U6

|  |  | Sequence |
| --- | --- | --- |
| miR-184 | Sense | 5’ TGGACGGAGAACTGATAAGGGT3’ |
|  | Antisense | ---- |
| U6 | Sense | 5’ CTCGCTTCGGCAGCACATATA3’ |
|  | Antisense | ---- |
