# Supplementary material for: Candidate tumour suppressor CCDC19 regulates miR-184 direct targeting of C-Myc thereby suppressing cell growth in non-small cell lung cancers
Source: J Cell Mol Med. 2014 Jun 26;18(8):1667–79. doi: 10.1111/jcmm.12317 (PMC4190912; doi:10.1111/jcmm.12317)
Supplement: Supplementary file 9 — Table S4 Primer sequences for C-MYC CDS. [file jcmm0018-1667-SD9.doc]

Table S4 Primer sequences for C-MYC CDS

|  |  | Sequence |
| --- | --- | --- |
| C-Myc CDS | Sense | 5’ATCGCTCGAGTGCTGCCAAGAGGGTCAA 3’ |
|  | Antisense | 5’ATCGCGGCCGCCGTTTCCGCAACAAGTCC 3’ |
