# Supplementary material for: Candidate tumour suppressor CCDC19 regulates miR-184 direct targeting of C-Myc thereby suppressing cell growth in non-small cell lung cancers
Source: J Cell Mol Med. 2014 Jun 26;18(8):1667–79. doi: 10.1111/jcmm.12317 (PMC4190912; doi:10.1111/jcmm.12317)
Supplement: Supplementary file 10 — Table S5 ChIP Primer sequences for miR-184 promoter. [file jcmm0018-1667-SD10.doc]

Table S5 ChIP Primer sequences for miR-184 promoter

| miR-184 promoter |  |  |
| --- | --- | --- |
| Primer pair 1 | Sense | 5’ CACAACCCTGTAAATTTGG3’ |
|  | Antisense | 5’ AAGGTTAAGGGCTTGG3’ |
| Primer pair 2 | Sense | 5’ GGTTTCATTCCGATACAC3’ |
|  | Antisense | 5’ TGTCAGATTCTTCCGTCT3’ |
