# Supplementary material for: Candidate tumour suppressor CCDC19 regulates miR-184 direct targeting of C-Myc thereby suppressing cell growth in non-small cell lung cancers
Source: J Cell Mol Med. 2014 Jun 26;18(8):1667–79. doi: 10.1111/jcmm.12317 (PMC4190912; doi:10.1111/jcmm.12317)
Supplement: Supplementary file 11 — Table S6 Down-regulation of NESG1 protein in NPC compared to NP epithelium tissues. [file jcmm0018-1667-SD11.doc]

**Table S6 Downregulation of NESG1 protein in NPC compared to NP epithelium tissues**

| Group | Protein expression (n) | | | P Value |
| --- | --- | --- | --- | --- |
|  | Total | Low | High |  |
| Normal epithelium | 26 | 7 | 19 |  |
| Cancer | 73 | 38 | 35 | P=0.038 |
