# Supplementary material for: Candidate tumour suppressor CCDC19 regulates miR-184 direct targeting of C-Myc thereby suppressing cell growth in non-small cell lung cancers
Source: J Cell Mol Med. 2014 Jun 26;18(8):1667–79. doi: 10.1111/jcmm.12317 (PMC4190912; doi:10.1111/jcmm.12317)
Supplement: Supplementary file 12 — Table S7 Correlation between the clinicopathological characteristics and expression of NESG1 protein in lung cancer. [file jcmm0018-1667-SD12.doc]

| **Table S7. Correlation between the clinicopathologic characteristics and expression of NESG1 protein in lung cancer** | | | | |
| --- | --- | --- | --- | --- |
| Characteristics | n | NESG1 (%) | | *P* |
|  |  | High expression | Low expression |  |
| Gender |  |  |  | 0.294 |
| Male | 53 | 23(50.8%) | 30 (49.2%) |  |
| Female | 20 | 12(50%) | 8 (50%) |  |
| Age（y) |  |  |  | 0.484 |
| ≥60 | 38 | 20 (53.8%) | 18 (46.2%) |  |
| ＜60 | 35 | 15 (48%) | 20(52%) |  |
| Pathology classification |  |  |  | 0.342 |
| squamous cell carcinoma | 30 | 12(38.5%) | 18(61.5%) |  |
| adenocarcinoma | 43 | 23(40%) | 20(60%) |  |
| Differentiated degree |  |  |  | 0.408 |
| High | 12 | 8(36%) | 5(64%) |  |
| middle | 19 | 10(61.8%) | 9(38.2%) |  |
| Low or undifferentiated | 41 | 17(50%) | 24(50%) |  |
| T classification |  |  |  | 0.586 |
| T1+T2 | 63 | 29(45.1%) | 34(54.9%) |  |
| T3+T4 | 10 | 6(72.2%) | 4(27.8%) |  |
| N classification |  |  |  | 0.336 |
| N0+N1 | 45 | 24 (39.7%) | 21 (60.3%) |  |
| N2+N3 | 28 | 11 (71%) | 17 (29%) |  |
| Distant metastasis |  |  |  | 1.000 |
| Negative | 72 | 35 (100%) | 37 (0%) |  |
| Positive | 1 | 0 (48.8%) | 1(51.2%) |  |
| Clinical stage |  |  |  | 0.342 |
| I～II | 43 | 23(45.1%) | 20(54.9%) |  |
| III～IV | 30 | 12(72.2%) | 18(27.8%) |  |
