# Supplementary material for: Candidate tumour suppressor CCDC19 regulates miR-184 direct targeting of C-Myc thereby suppressing cell growth in non-small cell lung cancers
Source: J Cell Mol Med. 2014 Jun 26;18(8):1667–79. doi: 10.1111/jcmm.12317 (PMC4190912; doi:10.1111/jcmm.12317)
Supplement: Supplementary file 13 — Table S8 Summary of univariate and multivariate Cox regression analysis of overall survival duration. [file jcmm0018-1667-SD13.doc]

| Table S8．Summary of univariate and multivariate Cox regression analysis of overall survival duration | | | | | | | |
| --- | --- | --- | --- | --- | --- | --- | --- |
| Parameter | Univariate analysis | | |  | Multivariate analysis | | |
|  | *P* | HR | 95%CI |  | *P* | HR | 95%CI |
| Age |  |  |  |  |  |  |  |
| ≥60vs. ＜60 years | 0.146 | 0.605 | 0.307-1.192 |  | 0.043 | 0.462 | 0.219-0.975 |
| Gender |  |  |  |  |  |  |  |
| Male vs. female | 0.593 | 1.231 | 0.574-2.641 |  | 0.868 | 1.074 | 0.462-2.500 |
| Pathology classification |  |  |  |  |  |  |  |
| Squamous vs. Adenocarcinoma | 0.151 | 0.590 | 0.287-1.212 |  | 0.013 | 0.338 | 0.143-0.798 |
| Differentiation degree |  |  |  |  |  |  |  |
| High vs. Middle vs.Low | 0.375 | 0.809 | 0.507-1.292 |  | 0.419 | 0.804 | 0.474-1.364 |
| T classification |  |  |  |  |  |  |  |
| T_1_-T_2_ vs. T_3_-T_4_ | 0.487 | 1.402 | 0.541-3.636 |  | 0.570 | 1.376 | 0.457-4.142 |
| N classification |  |  |  |  |  |  |  |
| N_0_­-N1 vs. N_2--_N_3_ | 0.173 | 1.599 | 0.814-3.142 |  | 0.004 | 0.067 | 0.11-0.430 |
| M classification |  |  |  |  |  |  |  |
| M_0_ vs. M_1_ | 0.876 | 1.173 | 0.160-8.623 |  | 0.175 | 0.224 | 0.26-1.941 |
| Clinical stage |  |  |  |  |  |  |  |
| Ⅰ-Ⅱ vs. Ⅲ-Ⅳ | 0.059 | 1.914 | 0.975-3.760 |  | 0.002 | 18.352 | 2.940-114.541 |
| NESG1 expression |  |  |  |  |  |  |  |
| High vs. Low * | 0.016 | 0.419 | 0.206-0.851 |  | 0.003 | 0.294 | 0.129-0.665 |
